# Telesimulation about home visits and child care: facilitators, barriers and perception of Nursing students

**DOI:** 10.1590/1518-8345.6037.3672

**Published:** 2023-01-06

**Authors:** Aline Natália Domingues, Jeniffer Stephanie Marques Hilário, Débora Falleiros de Mello, Ana Isabel Parro Moreno, Luciana Mara Monti Fonseca

**Affiliations:** 1 Universidade de São Paulo, Escola de Enfermagem de Ribeirão Preto, PAHO/WHO Collaborating Centre for Nursing Research Development, Ribeirão Preto, SP, Brazil.; 2 Bolsista do Conselho Nacional de Desenvolvimento Científico e Tecnológico (CNPq), Brazil.; 3 Universidade de São Paulo, Escola de Enfermagem de Ribeirão Preto, PAHO/WHO Collaborating Centre for Nursing Research Development, Departamento de Enfermagem Materno-Infantil e Saúde Pública, Ribeirão Preto, SP, Brazil.; 4 Universidad Autónoma de Madrid, Researcher (Cirugía), Madrid, Comunidade de Madrid, Spain.

**Keywords:** Pediatric Nursing, Child Care, House Calls, Computer Simulation, Nursing Students, Nursing Education, Enfermagem Pediátrica, Cuidado da Criança, Visita Domiciliar, Simulação por Computador, Estudantes de Enfermagem, Educação em Enfermagem, Enfermería Pediátrica, Cuidado infantil, Visita Domiciliaria, Simulación por Computadora, Estudiantes de Enfermería, Educación en Enfermería

## Abstract

**Objective:**

to evaluate the facilitators, barriers and perceptions of Nursing students in learning about home visiting and child care through Telesimulation during the COVID-19 pandemic.

**Method:**

a qualitative study to evaluate Telesimulation via computers, grounded on Kolb’s theoretical model. A semi-structured questionnaire and the Student Satisfaction and Self-Confidence in Learning Scale were applied, with descriptive analysis and qualitative thematic analysis on the perceptions of 41 Nursing students.

**Results:**

the contextualized Telesimulation provided learning opportunities in dimensions of the pedagogical strategy, telesimulated scenario, communication and specificities of child care in home visits. It was considered a safe and dynamic activity that helped knowledge consolidation and reflective attitudes, proximity to reality, and develop interaction, observation and types of approaches. There were restrictions due to Internet connection failures. A large percentage of the students indicated good satisfaction and self-confidence level with learning in the scale applied.

**Conclusion:**

the real clinical situation with remote immersion allowed observation, decision-making, reflection and elaboration of conclusions, inherent to the experiential learning cycle. The set of elements of this Telesimulation created an environment that stimulated the interest of Nursing students for other learning stages, suggesting a space that strengthens knowledge and maintains dialogue with face-to-face practices.

## Resumo

**Objetivo:**

avaliar as facilidades, barreiras e percepções de estudantes de enfermagem na aprendizagem sobre visita domiciliar e cuidado infantil por telessimulação na pandemia da COVID-19.

**Método:**

estudo qualitativo avaliativo de telessimulação por computador, fundamentado no modelo teórico de Kolb. Foram aplicados um questionário semiestruturado e a Escala de Satisfação de Estudantes e Autoconfiança na Aprendizagem, com análise descritiva e análise qualitativa temática sobre percepções de 41 graduandos de enfermagem.

**Resultados:**

a telessimulação contextualizada proporcionou oportunidades de aprendizagem em dimensões da estratégia pedagógica, cenário telessimulado, comunicação e especificidades do cuidado infantil em visita domiciliar. Atividade segura e dinâmica, auxiliou a solidificar conhecimentos e atitudes reflexivas, aproximação à realidade, desenvolvimento da interação, observação e tipos de abordagens. Houve restrições por falhas de conexão. Grande parte dos estudantes indicou níveis bons de satisfação e autoconfiança com a aprendizagem na escala aplicada.

**Conclusão:**

a situação clínica real com imersão remota permitiu observação, tomada de decisão, reflexão e elaboração de conclusões inerentes ao ciclo de aprendizagem experiencial. O conjunto de elementos desta telessimulação criou um ambiente que estimulou o interesse dos estudantes de enfermagem para outras etapas de aprendizagem, sugerindo um espaço que fortalece conhecimentos e que guarda interlocução com as práticas presenciais.

## Resumen

**Objetivo:**

evaluar las facilidades, barreras y percepciones de estudiantes de enfermería en el aprendizaje sobre visita domiciliaria y cuidado infantil por telesimulación en la pandemia de COVID-19.

**Método:**

estudio cualitativo que evalúa la telesimulación por computadora, basado en el modelo teórico de Kolb. Se aplicó un cuestionario semiestructurado y la Escala de Satisfacción de los Estudiantes y Autoconfianza en el Aprendizaje, con análisis descriptivo y análisis temático cualitativo sobre las percepciones de 41 estudiantes de enfermería.

**Resultados:**

la telesimulación contextualizada brindó oportunidades de aprendizaje en los aspectos estrategia pedagógica, escenario telesimulado, comunicación y especificidades del cuidado infantil en visitas domiciliarias. Es una actividad segura y dinámica, que contribuyó a consolidar conocimientos y actitudes reflexivas, permitió un acercamiento a la realidad, el desarrollo de la interacción, observación y tipos de acercamientos. Hubo restricciones por fallas en la conexión. La mayoría de los estudiantes indicaron buenos niveles de satisfacción y confianza en sí mismos con el aprendizaje en la escala aplicada.

**Conclusión:**

la situación clínica real con participación a distancia permitió la observación, toma de decisiones, reflexión y elaboración de conclusiones inherentes al ciclo de aprendizaje experiencial. El conjunto de elementos de esta telesimulación creó un ambiente que estimuló el interés de los estudiantes de enfermería por otras etapas de aprendizaje, por lo que se considera un espacio que fortalece el conocimiento y dialoga con las prácticas presenciales.

**Highlights**

(1) Innovation in the use of home visit telesimulation for Nursing students.

(2) Remote immersion in a real clinical situation allows observation, decision and reflection.

(3) Students with a high level of satisfaction regarding the telesimulation teaching resources.

(4) The environment stimulates the students’ interest in other learning stages.

(5) Telesimulation dialogues with the in-person practices.

## Introduction

Remote education became essential given the physical distancing sanitary measures and the suspension of in-person pedagogical activities during the COVID-19 pandemic^1^ ^-^ ^4^. 

Adaptations of learning scenarios and processes were carried out during the period of the aforementioned pandemic, adopting Telesimulation based on immersive real cases to provide innovative opportunities through telecommunication and simulation resources^1^ ^-^ ^2^ ^,^ ^5^.

Telesimulation is a field in evolution, with emphasis for clinical evaluators to promote competencies in undergraduate students^2^ ^,^ ^6^. Telesimulation seeks to imitate, from a distance, the particularities of a given context, to reach an understanding of the real conditions, using an environment set up to recreate a reality, with the purpose of practicing, learning, testing and evaluating^7^. 

The COVID-19 pandemic context, with the adaptations for undergraduate teaching^1^ ^-^ ^2^ ^,^ ^5^ and the relevance of pedagogical simulation activities, from a controlled space for the exchange of knowledge, procedures and interpersonal communication^2^ ^,^ ^8^, motivated conduction of the current research with a focus on learning opportunities for Nursing students about Home Visits (HV) in child care in a safe environment. HV is considered a mechanism for the provision of prevention, promotion and intervention services at homes, which, in general, is carried out by Nursing to address sustainable practices that support the improvement of health, well-being and developmental outcomes of children and families^9^. 

Thus, this study aims at assessing the facilitators, barriers and perceptions of Nursing students in learning about HV and child care through Telesimulation in the COVID-19 pandemic. 

## Method

### Study type

A qualitative and evaluative study of a computer Telesimulation activity on HV and child care, based on Kolb’s theoretical model of the experiential learning cycle^10^. The study followed the aspects listed in the Consolidated Criteria for Reporting Qualitative Research (COREQ)^11^.

The experiential learning cycle involves the students immersing themselves in a concrete experience (simulated scenario) to observe, decide what to do, reflect on themselves and others (debriefing), re-evaluate the experiences and draw conclusions or inductive systematic abstractions, in order to refine frameworks for the clinical practice and empirically test action plans^10^.

### Study locus 

A research study carried out in a virtual environment, using the Google Meet^®^ and Google Forms^®^ platforms, with undergraduate Nursing students from a public university located in Ribeirão Preto-SP, Brazil, focusing on a didactic activity of an undergraduate course in Nursing. 

### Period

The study was developed between May 26^th^ to October 27^th^, 2021.

### Selection criteria

Nursing students enrolled in the Bachelor’s Degree and Undergraduate Degree in Nursing (five years) of the aforementioned higher education institution were selected, together with the Comprehensive Health Care II (210 hours) discipline, in the 2021 academic year. Inclusion of the students was linked to participation in the remote session, in the Telesimulation activity about HV and child care, during the academic discipline and through the Google Meet^®^ virtual videoconferencing platform.

### Participants

The initial population consisted in 53 students attending third year of the undergraduate course enrolled in the aforementioned academic discipline, of which seven did not participate in the activity. There was no refusal to participate in the research. During the remote activity, five students withdrew their participation and seven did not attend the discipline the date in which this activity was conducted. The convenience sample was, 41 participants at both moments in Google Meet^®^ and Google Forms^®^.

### Process to design the Telesimulation

The theme of HV, which makes up the syllabus of the aforementioned discipline, was chosen for its relevance to child care and to integrate the Nursing practices in the field of primary health care^12^ ^-^ ^13^. With the suspension of face-to-face classes at the university, the simulated scenarios, originally planned, were reformulated and adapted to the new academic situation, via remote access and synchronous activities. Consequently, the topic proposed was organized in a Telesimulation process. Subsequently, at other moments of the academic discipline, the topic was also offered to the participating students as an in-person practice in health services.

The activity consisted of a single Telesimulation lasting two hours and thirty minutes, with one hour and ten minutes of prebriefing, twenty minutes of telesimulated scenario and one hour of debriefing. The same facilitator conducted all the activities proposed. 

The process established a safe learning environment, grounded on recommended practices^14^ and on the standards of simulation design best practices^15^. In addition to that, three attributes of the learning environment were preserved: ability to make mistakes without consequences, qualities of the facilitator and activity with guidance, preparation, objectives and expectation^16^. 

The prebriefing included an online conference, conducted by a professor in the field of Child Health Nursing, prior to the telesimulated scenario activity, to offer content relevant to the topic, as well as an initial explanation of the activity. 

The Telesimulation intervention was based on monitoring infant growth and development of a nine-month-old baby without special health needs. The setting was conducted with real data, based on the home a child and his reference parental caregiver. The approach was focused on specific aspects of child health, featuring a telesimulated home visit, centered on dialogue and observation, resolution of doubts and professional support regarding follow-up of the child’s growth and development, including visualization of real data from the child’s handbook and guidelines related to the COVID-19 pandemic context (protection measures, social distancing and vulnerable situations). 

The Telesimulation involved the participation of two actors, the baby and the grandmother, two teachers with availability of an operative microphone and webcam during development of the clinical case, with a computer screen and, simultaneously, the students participating in the activity. Two of them interacted with the actors, and the other students watched and listened, as in a typical simulation session. There was also the participation of two female graduate students, assisting in visualization of the written messages, supporting the students and professors, and making the link to the form available.

After the telesimulated home visit, the debriefing was carried out, permeating the emotional phases (How did you feel doing this activity), descriptive (How do you describe the scenario experienced?), evaluative (What did you do properly? What points could be improved?), and conclusive (What will you take from this to add to your learning?). Concomitantly, there was a theorization chain, highlighting aspects of communication with the parental caregiver; observation of the child; interaction with the child; specific elements of infant development and family relationships; parameters of infant growth, immunization, nutrition and sleep; guidelines for care routines and protection and safety measures in daily home life; and focus on comprehensive care. 

### Instruments

After the prebriefing, telesimulated scenario and debriefing, a semi-structured questionnaire was applied, containing open questions about facilitators and barriers of Nursing students, and the Student Satisfaction and Self-Confidence in Learning Scale (SCLS)^17^, through Google Forms^®^.

The questionnaire consisted of information on age, use of technological resources (computers, tablets, smartphones), Internet access tools and modalities used in academic activities, and participation in undergraduate simulation activities. After assessing the facilitators and barriers about the Telesimulation activity, it was also decided to record the students’ perceptions. The perceptions were obtained from two open questions: “What positive aspects would you like to highlight as a learning opportunity in the HV Telesimulation and child care activity?”, and “What negative aspects would you like to mention about learning in the HV Telesimulation and child care activity?” 

The SCLS scale presents 13 items with five-point Likert-type answers, where one point is the lowest satisfaction level and five points represent the highest satisfaction level^17^. Validation of the scale for the Portuguese language has psychometric properties with good potential for using the instrument, although there are limiting factors, such as sample size and its specificity^17^. 

### Data collection

Data collection was performed remotely, where the first contact was made via email. After the Telesimulation activity, a link for online access via Google Forms^®^ was made available. The form for online filling consisted in the characterization questionnaire, two open questions and the items from the SCLS scale.

### Data analysis

In the descriptive statistics analysis, the relative frequency of the variables investigated related to the results of the SCLS scale was calculated. Reflexive thematic analysis was used to analyze the students’ perceptions, with initial familiarization with the data, code generation, naming of topics and reporting^18^.

The SCLS scale presents 13 items with five-point Likert-type answers, where one point is the lowest satisfaction level and five points represent the highest satisfaction level^17^, presented in Figure 1.

**Figure 1:** Items from the SCLS scale^17^ used to evaluate the HV Telesimulation. Ribeirão Preto, SP, Brazil, 2022

Student Satisfaction and Self-Confidence in Learning Scale	
Item	Answers
1.	The teaching methods used in this simulation were helpful and effective.
2.	The simulation provided me with a variety of learning materials and activities to promote my learning the curriculum about home visits in children’s health.
3.	I enjoyed how my professor taught through the simulation.
4.	The teaching materials used in this simulation were motivating and helped me to learn.
5.	The way my professor taught through the simulation was suitable to the way I learn.
6.	I am conﬁdent that I am mastering the content of the simulation activity that my professor presented to me.
7.	I am conﬁdent that this simulation included the necessary content to master the curriculum of home visits in children’s health.
8.	I am conﬁdent that I am developing skills and obtaining the required knowledge from this simulation to perform the necessary procedures in a clinical setting.
9.	My professor used helpful resources to teach the simulation.
10.	It is my responsibility as a student to learn what I need to know through this simulation activity.
11.	I know how to get help when I do not understand the concepts covered in the simulation.
12.	I know how to use simulation activities to learn skills.
13.	It is the professor’s responsibility to tell me what I need to learn on the theme developed in the simulation during class time.

The procedures consisted of indexing information regarding the answers to open questions, whose codes capture an aspect and display a facet, and the thematic units add multiple facets and dimensions^18^. This process made it possible to highlight thematic units, which allowed analyzing opinions, attitudes, values and beliefs, avoiding a passive and decontextualized channel about the participants’ answers^18^ ^-^ ^19^. 

### Ethical aspects

The research was approved by a Research Ethics Committee, according to the recommendations set forth in Resolution 466/12, under opinion number 4,601,663.

The proposal was appraised and approved by the institution’s graduation commission. The students were recruited through electronic correspondence, according to the email address made available via USP webmail, detailing the research purpose and sending the research project and the Free and Informed Consent Form attached.

The participants were assigned names of gems as codenames. It is worth noting the absence of any authority relationship between students and professors.

## Results

The mean age of the students was 23 years old, with a minimum of 20 and a maximum of 40. The vast majority used notebooks (90.2%, n=37) and cell phones (95.1%, n=39) for the academic activities. The most used tools are Google Meet (100%, n=41), WhatsApp (90.2%, n=37), Google Drive (87.8%, n=36) and Zoom (29.3%, n=12), for participation in meetings, classes, group work and file sharing, among others. 

As for the simulation activity during Nursing undergraduation, most of them had already participated (97.6%, n=40), with Telesimulation seen as an interesting (85.4%, n=35), uninteresting (9.8 %, n=4) and indifferent (4.9%, n=2) opportunity.

The results emerged from data systematization, in a process for the apprehension of aspects about Telesimulation via computers on HVs and child care. Figure 2 presents the thematic units, the dimensions and the codes related to the Telesimulation for undergraduate Nursing students.

**Figure 2:** Presentation of the thematic units, dimensions and codes related to the HV Telesimulation. Ribeirão Preto, SP, Brazil, 2022

Thematic Units	Dimensions	Codes
Telesimulation: immediate and prospective learning	Pedagogical strategy	-Safe performance; -Content fixation; -Critical and reflective attitudes; -Dynamic strategy; -Connection failures
Context integrated to the house: the perspective about Telesimulation	Telesimulated setting	-Proximity to reality; -Real situations; -Training; -Different and interesting
Interaction in the HVs: barriers and facilitators	Communication	-Which approaches to use; -What to ask and watch; -Dealing with socialization; -Shame about participating
HVs in children’s health revitalized by Telesimulation	Specificities of children’s care	-Contact with an infant; -Infant development; -Context where the child lives; -Concepts related to the infant’s health

### Telesimulation: immediate and prospective learning

This theme portrays Telesimulation in its pedagogical strategy dimension, with elements that favor the moment itself and, simultaneously, shed light on the next stages of the learning process. 

It represented an opportunity to strengthen knowledge: *The explanation and then the practice in the form of Telesimulation consolidated knowledge (Tourmaline); Imagining the real situation is very striking and learning becomes more fixed (Topaz); Content fixation through the practical simulation activity was positive, the group’s contribution in the elaboration of ideas, and proximity to reality in this pandemic moment* (Pyrite)*.*

It distinguished itself as an aggregator of new explanations: *The knowledge acquired added a lot of new information (Unakite); It is an opportunity to analyze how we should behave as future professionals and, as it is a simulation, it generates greater self-confidence (Jade); It was possible to experience in the practice how a Telesimulation, a dynamic strategy, draws the students’ attention (Emerald).*

It was indicated as an opportunity for reflection: *The new, the challenge, it is a positive aspect, so that the students can have critical and reflective thinking and attitudes (Coral); Viewing as a spectator and seeing the situation as a whole made me be more critical of some of the points discussed (Tiger’s Eye); The possibility of performing safely and understanding how barriers occur in Teleservice (Ruby).*

These opportunities bring about positive points that denote interest in the content and approach, with openness to the diverse information worked on that configure immediate learning and generate prospective interest.

On the other hand, the limits of the remote activity were perceived: *The unstable Internet connection, I often lost what the interviewer or interviewee was saying and this sometimes caused my reasoning to be lost (Diamond); A few connection failures affected understanding of some sentences a little, but these are expected aspects in a virtual environment (Crystal); For me, Telesimulation is not as close to reality as face-to-face simulation, I feel that it is not as effective, that is, connection problems get in the way, distractions happen (Unakite).*

Telesimulation was seized in its favorable, viable and restrictive points, as an opportunity to reach knowledge, which allowed access in the pandemic context. There were some gaps due to interruptions in the Internet connection, which may have interfered in chaining of the ideas.

### Context integrated to the house: the perspective about Telesimulation via computers

This theme brings to light Telesimulation in its dimension of a telesimulated setting, pointing out aspects that express, in a way, a triangulation of integrated contexts, between the students’, the teachers’ and the actors’ homes. 

Online immersion allowed integration to acquire factual aspects: *The HV itself was positive, with real people and real situations (Grenade); Watching how conversations can be carried out when they involve adults, children and professionals, discussing points that can be addressed at another time and those that are important in this first contact (Hematite).*

The perspective adopted recorded a real context, representing an opportunity marked by proximity to reality: *We got closer to reality, even if remotely, training for when we go to do practical activities in the field (Onyx); The possibility of a contact with reality, the online HV, and the opportunity to acquire knowledge about other subjects were very positive (Opal).*

The exercise of establishing contact with a contextualized environment, and somehow expanding a real situation, requires that students get involved and value attention to the activity, as a direct result of their efforts in the learning process. 

On the other hand, it is acknowledged as an activity marked by distance: *Due to adequacy of the moment, I believe it created some distance, but the activity had a different and interesting proposal (Sapphire); Difficulty following all the dynamics of the visit due to instability of the Internet (Hematite).*

The immersive remote nature of Telesimulation provided opportunities for learning from a situation tangent to reality, particularly to observe, reflect, reevaluate and elaborate syntheses. Telesimulation is seen as an alternative and requires resources to prevent distractions and interruptions.

### Interaction in the HVs: barriers and facilitators

This topic addresses Telesimulation in the communication dimension, showing interaction possibilities in the activity performed.

The singularities to establish contact and to obtain and transmit information were seen as positive: *The opportunity for us to think about elements that we might ask about, which approaches we could use to establish effective communication with the child’s caregiver (Agate); It guided what would be important to ask and watch about the context where the child lives (Amber); It made me reflect and analyze how I could address a HV, or what to ask and analyze, as well as suggestions I could make for a possible improvement in the health situation faces at that moment (Turquoise).*

Some aspects related to conduction of the HVs were contemplated: *The Telesimulation was important for us to get an idea of what a HV is like (Aquamarine); Watching the way the girls asked the mother about the child and the problems they may have and that influence her health (Turquoise); Being able to simulate a HV allows students more confidence when making visits, and Telesimulation greatly enriches learning (Diamond); Development of anxiety control, of the conversation, coping with socialization (Quartz).*

On the other hand, intercommunication did not flow so well throughout the activity: *Many times people may feel ashamed about participating via Google Meet, which ends up in a less dynamic class (Turquoise).*

Establishing communication is a process based on exercising interaction through dialog, reflecting on how to conduct it and deal with obstacles in conversation. Telesimulation offers an interesting moment due to the short-term effects related to the time to explore communication and the approach performed.

### HVs in children’s health revitalized by Telesimulation

This topic exposes Telesimulation in the dimension of the specificities of HVs aimed at child care, showing characteristics of the child and the relationship with the parental caregiver. 

Some elements demarcate contact with the child’s health: *I was able to better understand how to manage a HV with a child (Moon Stone); Having more contact with a baby, knowing more about it, inserting myself in this reality (Jasper); Rescuing important concepts for the baby’s health (Citrus).*

The perceptions about child development and the family context were pointed out: *Being able to listen to a family member talk about development of the child was very enriching, as well as addressing a number of aspects discussed in the classroom (Pearl); Having a critical perspective about the diverse information reported by the grandmother, not only focused on the baby’s issues, but also on the family context (Citrine).*

The addition of the activity in a real situation with a child offers knowledge exchange, with stimulation and visualization through the screen and apprehension of the parental caregiver role. These aspects arise criticality for the moment to discuss what was captured on the scene.

On the other hand, gaps in immersion refer to the need to implement actions with the child: *The lack of physical contact is a negative aspect (Pyrite); Lack of human proximity, examination of measurements on the baby, aspects of the child that could be verified closer if it were in person (Quartz); Failing to perform the physical examination (Topaz).*

The distance imposed by the telesimulated activity deals with technological access, its limits and an imaginary construction of some points of the HV experienced. 

There are indications of the continuity of opportunities in the learning process: *There are difficulties, but everything is learned over time (Coral); There were no negative aspects, it only increased my desire to go to the field and be able to carry out the visit in person (Agate); Obviously everything is better when it is done in person, eye to eye, visiting the home, etc.; however, given the moment we are living, Telesimulation was effective and fulfilled its role (Jade).*

Telesimulation offered learning opportunities to the students, particularly through dialogue, observation and reflection about a clinical case involving children’s health. It is innovative and creative because it opens up possibilities to retake elements of care at a later moment, establishing continuity bridges with the face-to-face practice to advance, especially in interventions. 

**Figure 3:**
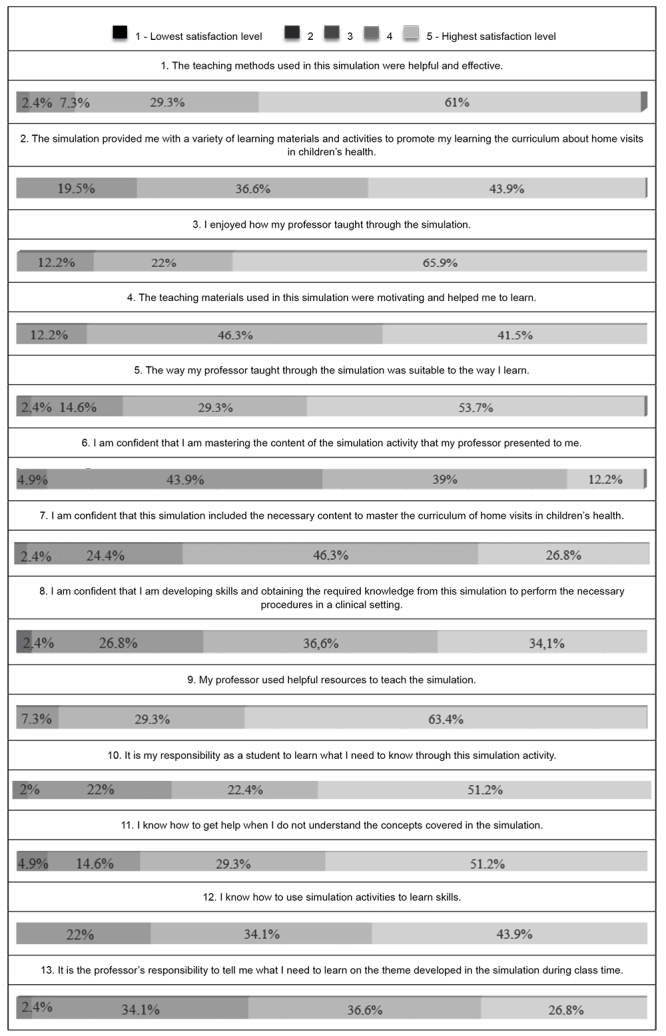
SCLS results for undergraduate Nursing students. Ribeirão Preto, SP, Brazil, 2022

Regarding the SCLS levels, Figure 3 presents the results of the scale for undergraduate Nursing students.

It is noted that a large percentage of the students pointed out good levels (scores of four and five). There was a higher level of satisfaction regarding the methods and resources used, conduction of the Telesimulation, availability of motivating materials, obtaining the necessary knowledge from the Telesimulation, and the student’s responsibility to learn and to seek help.

The aspects pointed out with the lowest level of satisfaction were related to confidence to master the child’s health content and the professor’s responsibility to say what the students need to learn in the theme developed during the telesimulated activity. 

## Discussion

The results of the current study present facilitators, barriers and perceptions expressed by Nursing students regarding learning opportunities through contextualized Telesimulation, during the COVID-19 pandemic. Most of the students considered Telesimulation as an interesting opportunity. The dimensions found are linked to the pedagogical strategy, to the telesimulated setting, to communication, and to specificities of child care in HVs. The favorable elements of a safe and dynamic activity stand out, which helps consolidate knowledge and reflective attitudes, with proximity to reality, development of interaction, observation and types of approaches, as well as the appropriation of aspects of the conduction of HV in child care. Restrictive points were recognized, due to Internet connection failures, affecting the breadth of activity monitoring, as well as gaps in more direct interaction with the child. 

The use of Telesimulation to teach a complex theoretical-practical scenario based on cases is relatively new, as well as working with all students in their homes, as it involves providing an authentic and immersive mode, with a real case and opportunity to practice applied actions in the clinical field^1^. In this sense, in the current research, the telesimulated scenario brought to light aspects of triangulation of contexts, seeking to integrate the actors’ house, including presence of the child and the parental caregiver and the interaction, with the students’ and the teachers’ houses. The benefits of Telesimulation extend beyond simulation centers and it is useful where distance limitations prevent effective and efficient instruction of a particular practice^20^.

The COVID-19 pandemic context has generated changes in the everyday life of teaching, with Telesimulation being an alternative aimed at continuing undergraduate education and creating robust remote educational experiences that maximize learning opportunities^1^ ^-^ ^4^ ^,^ ^21^ ^-^ ^22^, similarly to that found in the current study. In Brazil, during the COVID-19 pandemic, not only did the education area undergo changes in its routine, the health area in turn issued programmatic guidelines for primary health care related to the reorganization of actions, for example, the routine vaccination service was postponed at times throughout the national territory and teleservices were introduced, as measures to reduce face-to-face contact. Based on documentary analysis, a study identified that longitudinality of the health actions was affected by the reduced access to child health promotion, during the COVID-19 pandemic^23^.

Regarding the perception about Telesimulation, most of the students indicated satisfaction, a result also identified in other studies, which found greater engagement and stimulus for critical thinking^1^ ^,^ ^23^, flexibility and facilitations in the discussion^24^, as well as to learn both by observing and actively engaging in the activity^25^. 

In addition, several studies pointed out limitations of remote activities, due to gaps in audio quality^1^, distractions and limited Internet connection^3^, similarly to the current research.

Mastery of content and skills related to children’s health can be increased with interventions in face-to-face HVs in child health, advocated in the scientific literature for its far-reaching benefits in favor of healthy growth and development in early childhood and to parental caregivers, who need support to build home environments capable of providing care and protection^12^ ^-^ ^13^ ^,^ ^26^. The Telesimulation with the theme of HV in child health proved to be viable, bringing about a contribution to teaching-learning about interviewing the parental caregiver, and observation and analysis of child care at home, which reserve articulations with the following stages of the discipline.

It is worth pointing out that the Nursing students’ desire to learn more with in-person HVs is legitimate.

Telesimulation appears as a possibility to redesign a practice and offers a set of elements that are likely to converge pedagogically in the name of meaningful learning^27^. Thus, it is a practice that reveals aspects that can be worked on in advance, reinvigorates students’ interest in learning and, as an active guide, opens up possibilities for other learning stages.

Elaboration of a situation tangent to reality to provide the apprehension of factual aspects was relevant in the current study and, from this influx, other perceptions were born, provided by the debriefing moment, for the construction of visions of the professional practice and health care. Debriefing is considered effective in providing experiences and opportunities for acquiring knowledge and has been used in simulation in Nursing education to improve clinical skills and learning outcomes^28^. 

The experiences that Nursing students have with experiences and use of technologies during graduation are seen as a possibility of training and proximity to Telehealth, which is growing in the clinical practice and linked to the increase in health care^29^ ^-^ ^30^.

The limitations of the current study refer to the apprehension of elements about Telesimulation centered on the students and on data collection in writing, suggesting the expansion to different participants and at different times, in future research studies.

Given the COVID-19 pandemic context, remote education has become predominant, even in health courses, and the identification and analysis of facilitators, barriers and perceptions by students about learning through Telesimulation favor attaining solutions and the improvement of the professors who conduct these activities, also improving the objects that facilitate knowledge. 

## Conclusion

In the current research, the opportunities limited to Telesimulation on the theme of HV and child care highlight the facilitators, barriers and perceptions of this type of activity. The facilitators are centered on positive communication, details regarding conduction of the telesimulated activity, the realistic context, knowledge reinforcement, and reflection. The barriers are focused on the problems related to the Internet and on gaps in the actions most targeted at the children. The perceptions express the students’ satisfaction with Telesimulation, which, through the SCLS scale, show higher levels of satisfaction in the method and resources used, motivating materials and the students’ responsibility to seek other contributions and knowledge.

Telesimulation involved remote immersion, observation, decision-making, reflection and drawing up conclusions from a real clinical situation, processes inherent to the experiential learning cycle. A set of elements created an environment that stimulated the interest of Nursing students for other learning stages, suggesting a space that strengthens knowledge and maintains dialog with face-to-face practices.

Telesimulation is a promising pedagogical strategy, with sufficient creativity to offer the students diversified ways of learning. By preserving a safe and realistic environment, there are limits and opportunities to develop interaction skills, clinical judgment and decision-making, as well as to apply therapeutic communication, in order to compose comprehensive health care. 

Opportunities during undergraduation that are similar to Telehealth, based on Telesimulation that ensure satisfactory, self-confident and relevant situations for the professional practice, can favor a fruitful theoretical-practical learning process. 
